# Mobile health apps for pregnant women usability and quality rating scales: a systematic review

**DOI:** 10.1186/s12884-023-06206-z

**Published:** 2024-01-05

**Authors:** Mohammad Reza Mazaheri Habibi, Fateme Moghbeli, Mostafa Langarizadeh, Seyed Ali Fatemi Aghda

**Affiliations:** 1 Department of Health Information Technology, Varastegan Institute for Medical Sciences, Mashhad, Iran; 2 Department of Health Information Management, School of Health Management and Information Sciences, Iran University of Medical Sciences, Tehran, Iran; 3 Research Center for Health Technology Assessment and Medical Informatics, School of Public Health, Shahid Sadoughi University of Medical Sciences, Yazd, Iran

**Keywords:** Reproduction, Women, mHealth, Telemedicine, eHealth, Nielsen principles

## Abstract

**Objective:**

This study is to identify the apps used by pregnant women during the technology era and to choose the best app from the point of view of pregnant women and experts.

**Methods:**

The article is a research article that uses PRISMA flowchart. Given that there are many apps in the field of pregnancy and due to technological advances, the articles of the last 13 years that have been scientifically published in the databases of Google Scholar, PubMed, and Science Direct have been analyzed. The most widely used and, at the same time, the best app is introduced in terms of its high usability in users’ attitude. Finally, Apps will be compared in terms of accuracy, precision, and usability of the dimensions of Jacob Nielsen's five principles.

**Results:**

According to the search strategy, 23 articles were identified qualitatively by reviewing both authors. Then, the types of apps were divided into three general categories, pregnant entertainment apps, pregnant information apps, and monitoring apps for mothers' physical health. Finally, 10 apps were selected and the Amila app was introduced as the best due to its high usability (Effectiveness %66.66) and users’ satisfaction or women’s choice (%98).

**Conclusion:**

Using trusted apps to maintain their health and reduce traffic will be very important. Given that this research article was written with the aim of choosing the best app, that not only provides the required information to mothers, but also the ability to interact with doctors and specialists.

**Supplementary Information:**

The online version contains supplementary material available at 10.1186/s12884-023-06206-z.

## Introduction

In today's world of technology, electronic devices such as cell phones and tablets have become an essential tool in everyday life. Whether used for formal or personal purposes, these devices influence and shape the way people think and live. The rapid development of Internet-based communication technologies over the past twenty years has changed the lifestyles of people around the world. Residents of cities and rural can equally access the opportunities used by mobile devices [1–3].

When it comes to women's health and addressing important issues such as childbirth and pregnancy, the most important thing is to constantly monitor the health of the mother and fetus [4, 5]. In the past, due to unnecessary considerations due to ignorance, information about pregnancy and childbirth was usually done incompletely and with strange restrictions. Some articles even considered educating and informing people about this natural issue an ugly act [6–8] .

But fortunately, over time, extremist views have largely disappeared, so that today there are many tools to inform young women about the important issue of pregnancy and maintaining their health during this critical period [9–12] .

Today, with the significant advancement of medical technologies in the field of pregnancy and childbirth, the world of applications and websites has entered all aspects of human life, including pregnancy [13–15]. These days, it can be rarely seen a pregnant woman who is unaware of health status of her fetus [16]. Therefore, the category of motherhood has been and is a very lucrative and popular subject for launching startups in the world, so that if you look at virtual stores, you can encounter with a considerable variety of apps and websites in the field of motherhood [17–19].

Today, the use of apps is more popular than websites. As a result, most reputable sites in the field of pregnancy have designed apps that usually use tools such as estimating the time of delivery, calculating BMI, determining sex, calculating the required calories, scheduling the vaccine, searching for the baby's name, etc. in this program are included that can be easily used. The “Pregnancy and Childhood Calendar” is also one of the most popular sections of the applications, which provides accurate and complete articles from the first week of pregnancy to the 41st week of pregnancy [20–28] .

Today, from the topic of pregnancy health and counseling in this field to counseling for buying clothes, designing a room and preparing baby food, it is discussed and progressed in the context of applications. There are also many websites that work in this field and are a kind of app for young mothers [3, 21, 23, 29, 30] .

But because the multiplicity of these apps, choosing the best type has become difficult for pregnant women. The reliability of the software, its accuracy and precision, and finally how to use it is very important. However, the main concern is the quality of these programs. Therefore, there is a need to monitor the quality of information provided by these pregnancy programs. In the development of interactive mobile applications, one of the main factors that determine their success is satisfaction [31]. However, although many researchers have done research on mobile health-related applications, many of these studies are inaccurate.

On the other hand, despite heavy traffic, traveling is a dangerous act for pregnant women, and using the app can reduce their travel to some extent and provide them with the necessary information and measurement.

However, the quality, reliability and effectiveness of existing pregnancy programs have not yet been determined. Therefore, exposure to potentially harmful programs or participation in research with evidence-based mobile applications should be carefully considered, especially during pregnancy when women are more sensitive to external influences. In addition, unnecessary information and advice about lifestyle and health care can cause more anxiety and stress during pregnancy. Therefore, information on usability and effectiveness is very important for implementing new programs in maternal health care [6, 17, 18, 24, 26, 27, 32] .

In this research article, pregnancy apps will be categorized first and then they will be compared in terms of accuracy, precision and usability of the dimensions of Jacob Nielsen's five principles, it will be given users a detailed view to choose the best type of app.

## Materials and methods

**Fig. 1:**
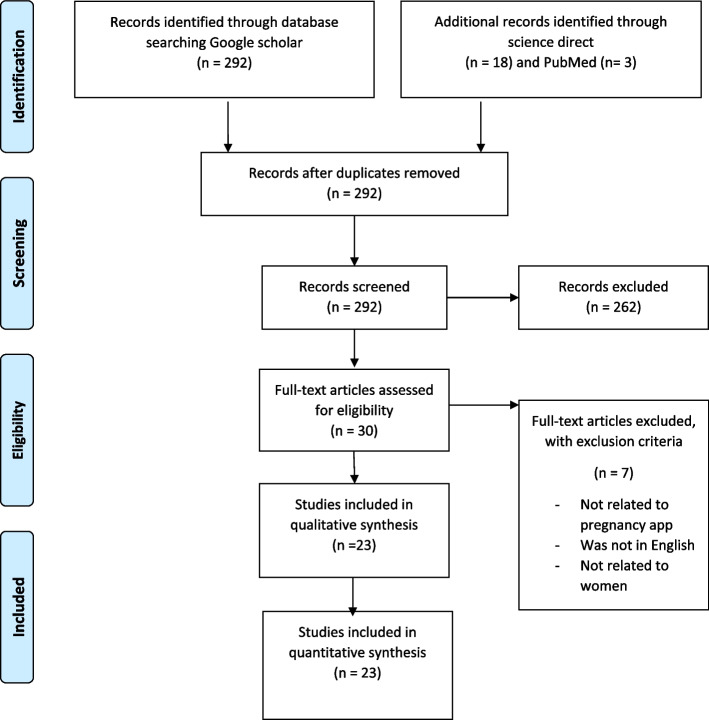
Article selection chart based on PRISMA flowchart

This article is a systematic review. The protocols for this article was registered to the PROSPERO database under the identification number CRD42023478584. First by searching for the keywords mHealth, pregnancy, mobile app, mobile phone, as well as combining the keywords in the Google Scholar, PubMed and Science Direct databases in the period October 2010 to September 2023, a total of 313 articles extracted. The strategy search was saved in Endnote version 20. Then, considering the inclusion and exclusion criteria (Table 1) by PRISMA flowchart and also reviewing the abstracts of articles by both authors separately, finally 23 articles were included in the study. Of these 23 articles, 10 apps were reviewed according to inclusion and exclusion criteria. Figure 1 shows the study selection chart.

**Table 1:** Inclusion and exclusion criteria of the study

Exclusion criteria	Inclusion criteria
Apps for fathers or plugins for fathers were excluded from the study	Articles in the period 2010–2023
Apps designed for pregnant women with a specific illness	Articles in English
Apps for caring for the mother and newborn baby after delivery	Access to the full text of the article
–-	Apps with the target group of pregnant women

On the other hand, the evaluation of apps information was validated by the authors according to the guidelines.

## Results

Of the 23 studies, 78% were conducted in Malaysia (29%), China (21%), the United States (19%) and Australia (9%). The apps reviewed in these articles included the dimensions of weight control, week-to-week care, pregnant women, and examining mothers' physical and mental condition, choosing a name for their child, and purchasing supplies. Table 2 shows the categories of different dimensions of pregnancy apps. There was a total of 251 apps on Google Play related to pregnant women, only a fraction of which have been scientifically reviewed, which is actually done in Table 2 of this category.. Google Play was checked for extracting users’ satisfaction. One of the most important parts of the app category is the information section. The information must be up-to-date and credible, and on the other hand, it must consider all sections of the pregnant woman and the scientific texts must be expressed in simple language according to guidelines. On the other hand, it is one of the sub-sections of information about specific pregnancies, which includes women with certain diseases such as diabetes, asthma and hypertension, which were excluded from the present study.

**Table 2:** Categories of pregnancy apps [1, 3, 6–9, 11–13, 16, 17, 23, 24, 26–29, 33–37]

Hobby	Monitoring	Information
-**Games**	-**Tracking weight and waist**	-**Information about pregnancy,**
-**Pregnancy test and ultrasound pranks**	-**Measurements**	-**Information about nutrition and exercise**
-**Shopping for pregnancy**	-**Diet**	-**Behaviors that should be avoided by pregnant women**
-**Gender predictors**	-**Water consumption**	-**Online forums in which to connect to other pregnant women (for example, to share and compare stories and experiences)**
-**Baby name generators**	-**Symptoms**	-**Recognize the time of baby birth**
-**Writing diaries**	-**Moods**	-**Special pregnancy (for ill mom)**
-**Training yoga**	-**Medications**	
-**Pregnancy calendar**	-**Cravings**	
	-**Appetite**	
	-**Energy levels**	
	-**Input due dates and appointments**	
	-**Production of a repository of personal medical information**	
	-**Monitoring every week (1st -41st)**	
	-**BMI**	

On the other hand, identifying the quality of apps also requires examining quality indicators such as usability (Table 3). Table 4 classifies the quality of pregnancy apps based on the dimensions of Nielsen's five principles of accuracy and precision based on literature review.

**Table 3:** Nielsen's Five Principles of Usability [38, 39]

The Jakob Nielsen’s usability principles
Effectiveness: easy to recover from errors
Efficiency: efficient to use
Learnability: easy to learn
Memorability: easy to remember
Satisfaction: subjectively pleasing

**Table 4:** The quality of apps based on Nielsen principles, accuracy and precision apps use extracted from articles

	**Apps’ name**	**Effectiveness**	**Efficiency**	**Learnability**	**Memorability**	**Satisfaction**	**Accuracy**	**Precision**
1	**Amila**	66.66%	77.14%	73.33%	76.66%	98%	98%	98%
2	**Day by Day Pregnancy Tracker**	75%	75%	75%	76%	75%	96%	96%
3	**Pregnancy + tracker**	75%	75%	75%	75%	75%	96%	96%
4	**Pregnancy & Baby Tracker**	75%	75%	75%	75%	75%	96%	96%
5	**Pregnancy + **	75%	75%	75%	75%	75%	96%	96%
6	**The Bump—Pregnancy & Baby Tracker**	75%	75%	75%	75%	75%	96%	96%
**7**	**Sprout Pregnancy**	85%	83%	75%	82%	88%	92%	92%
**8**	**Ovia Fertility & Cycle Tracker**	75%	75%	75%	75%	75%	96%	96%
**9**	**WebMD Pregnancy**	88%	88%	81%	83%	90%	90%	90%
10	**My Baby’s Movements app**	52%	53%	52%	62%	50%	56%	56%

**Fig. 2:**
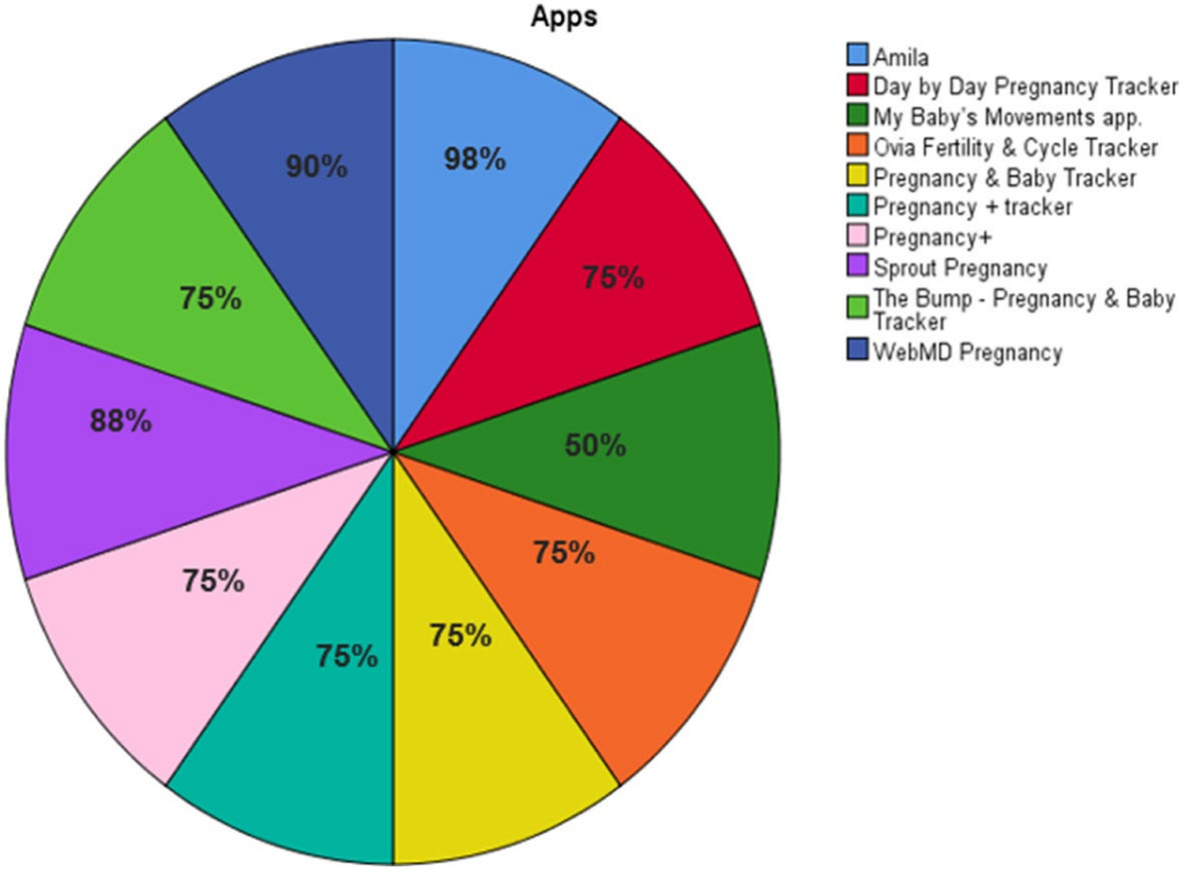
Satisfaction chart of pregnancy app users

Figure 2 shows a graph of user satisfaction with pregnancy software based on reports of app satisfaction on Google Play and the App Store.

Based on Table 4. Amila app has the highest users’ satisfaction in comparison with the other apps. And actually, has the better precision among the others.

### Amila app

The Amila Pregnancy mobile app has changed the delivery of healthcare services to pregnant women around the world that are increasingly beneficial in their daily lives. Only a few digital interventions have been developed for pregnant women, and little is known about the acceptance and use of such mobile apps that help pregnant women. According to the evaluation of this program, Nielsen usability criteria were obtained with an average of 71.824 percent. This program has the following capabilities that are more accurate than similar apps and are more satisfied with users [13] .

- Track your pregnancy week by week.
- Get information about your baby.
- Calculate current week of pregnancy.
- Calculate due date (pregnancy date).
- Track your weight.
- Track baby kicks.
- Make notes with your pregnancy symptoms (morning sickness, changes with your body, doctor appointment).
- Free pregnancy app.

**Fig. 3:**
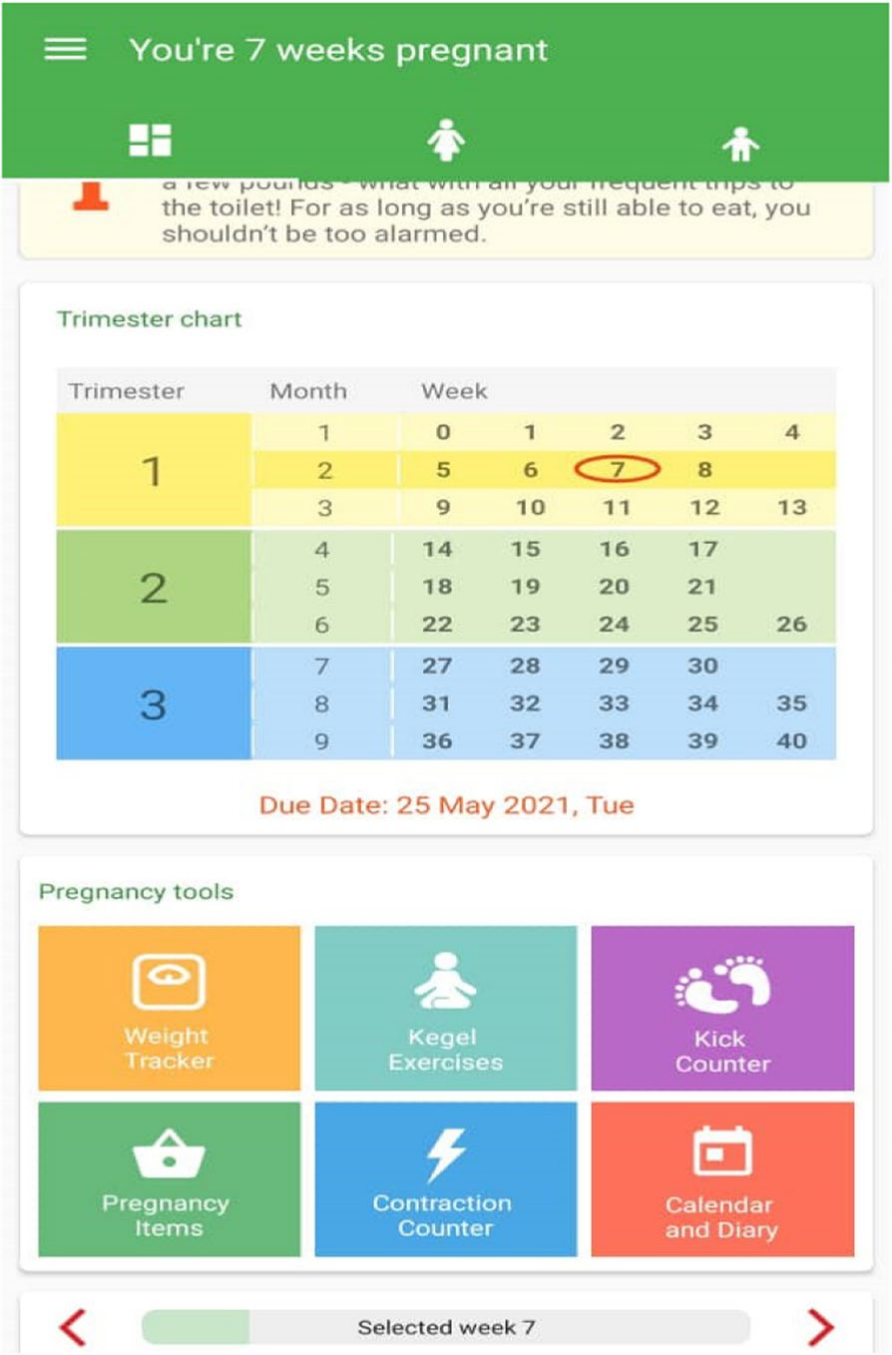
Amila App scheme

Figure 3 shows part of this software. As shown in the figure, the menu of this app has become more attractive in the 2023 update and has provided the ability to access different sections more quickly. Important parts of this software include weight monitoring, pregnancy exercises such as pregnancy yoga, weekly pregnancy information, and communication with the doctor, exchanging views with other mothers and using the experiences of pregnant women, pregnancy calendar and taking notes in it [8, 13].

### WebMD pregnancy

Five comprehensive tools to keep track of important needs and stay on schedule:

- Breastfeeding, bottle-feeding, and solid-food trackers – record and review all your feeding and nursing sessions, including both breast milk and formula. It has the highest satisfaction after Amila app [40, 41].

### Sprout pregnancy

Sprout pregnancy has the 3^rd^ rank in satisfaction. Trusted and recommended by expecting families all over the world, Sprout Pregnancy guides you through every day of your pregnancy. It helps keep you organized and educated about the many exciting changes and developments happening in your body and for your growing baby [42].

### “Day to day pregnancy tracking”

Day to Day Pregnancy Tracking application is the most advanced, comprehensive and useful pregnancy tracking application in Google Play. It NEVER requires an active Internet connection. This application will accompany you in your great adventure which will take 280 days. That is the 2^nd^ popular app based on Table 4 [43].

### Pregnancy + tracker

Pregnancy + tracker has the 3^rd^ rank in this paper. This pregnancy app will keep users calm throughout their pregnancy. It will let you know what's happening with your unborn, week by week, from that positive pregnancy test to delivery [44] .

The most applicable apps based on Nielsen principles were explained.

## Discussion

In this systematic review article of category pregnancy apps and Amila app according to Table 4 in terms of usability, accuracy and precision received the highest score compared to other apps and was suggested as a reliable app to pregnant women and experts in this field. According to the evaluation of this program, Nielsen usability criteria were obtained with an average of 71.824%. Given that there are many pregnancy apps in the Apple Store and Google Play, finding the most reputable ones is very difficult and requires scientific research. Out of 23 extracted articles, we reached a total of 10 apps for which usability criteria were examined.

Mohan S and et al. has done an RCT. The key point of this paper was to evaluate the usability and quality of the “E-Midwife” mobile application for nurse-midwives, which concentrates on obstetric complications. The article has shown that the E-midwife mobile application has a high usability and quality which makes its usage effective and efficient. It is shown that these similar mobile apps could be used along with other educational strategy for educating the nurse-midwives [45] .

Anderson LN and et al. were shown the development and assessment a reliable scale for measuring mobile health (mHealth) credibility. In this paper Three scale dimensions were chosen: concern, character, and competence. Both referent source for mobile apps and app developer were significantly associated with perceived mHealth credibility [46] .

Azham Hussain et al. In Malaysia evaluated Amila using the Nielsen Five Principles, which found that some of the main menu extensions were difficult for the public to access, and it was difficult for users to access the calendar and record events in it. In the 2023 version, these problems have been fixed [36, 47] .

A study by Alicia A. Dahl et al. collected pregnancy apps with a weight control approach for pregnant women, which collected the apps by 2016 and reported the results. In this article, out of 87 apps surveyed, only six met the inclusion criteria. Of these apps, Day by Day Pregnancy Tracker, Pregnancy + tracker, Pregnancy & Baby Tracker, and Pregnancy + also met the inclusion criteria in this study [2] .

A review by Sanne B Overdijkink et al. also examines the life cycle of pregnancy apps with the approach of pregnant women with the disease. This study was excluded due to exclusion criteria in this article and only the apps introduced in this article were used in this article [48] .

In 2017, Tamar Krishnamurti et al. introduced the MPH Pregnancy App as the first pregnancy app based on behavioral decision research that provides and collects risk information related to preterm delivery. In the new update, satisfaction is 56%. And due to the similarities with other software and lacked some of the features of Table 2, was not selected for this study and did not enter the study. Because in this study, articles that had all the features of Table 2 were reported [49] .

In general, it can be concluded that due to the high volume of pregnancy apps and the lack of review of many of them in terms of usability, a research article to review such apps in terms of user-friendliness and user satisfaction is important for pregnant women and professionals in this field. Given that many apps in the field of pregnancy are commercial and there is no scientific article about them, so they did not participate in the present study [1, 20–22, 33, 50] which is one of the main limitations of this article and the strength of the article is that the apps reported in this article has been scientifically validated by experts.

Today, the use of smartphones has grown significantly and most people use mobile apps instead of websites. Due to the prevalence of different viruses and the risk of pregnant women, most pregnant women have turned to apps to reduce their travel to find out about their status [20, 21, 23–25, 27, 28, 51, 52]. But using a reputable and reliable app is also very important. Because a search on Google Play or the App Store shows a large number of apps for them, many of which may not be scientifically qualified [53–59] .

In this article, the best apps that are also popular from the users' point of view were introduced so that not only pregnant women but also obstetricians can use them. Some of the available apps were designed specifically for pregnant mothers. Mothers with underlying diseases such as diabetes, asthma and high blood pressure. Such apps were excluded from the study because they included a certain segment of pregnant women, and finally 10 of the most widely used apps in the field of pregnancy were selected, which were approved by users and scientifically evaluated.

Amila App, WebMD Pregnancy, Sprout Pregnancy, Day to Day Pregnancy Tracking and Pregnancy + tracker had the highest satisfaction range, 98%, 90%88%75% and 75% respectively. In comparison with other apps, the highest accuracy and precision are reported too. So, they can be introduced to doctors and pregnant women as a good app for tracking. Details of the included studies are shown in Table 5.

**Table 5:** Details of the included studies

Authors	Aim	Conclusion
Azham Hussain et al	evaluating Amila using the Nielsen Five Principles	found that some of the main menu extensions were difficult for the public to access, and it was difficult for users to access the calendar and record events in it
Alicia A. Dahl et al	collected pregnancy apps with a weight control approach for pregnant women	six met the inclusion criteria. Of these apps, Day by Day Pregnancy Tracker, Pregnancy + tracker, Pregnancy & Baby Tracker, and Pregnancy +
Sanne B Overdijkink et al	introduced the MPH Pregnancy App as the first pregnancy app based on behavioral decision research	satisfaction is 56%. And due to the similarities with other software and lacked some of the features of Table 2, was not selected for this study and did not enter the study
Fabrizio Bert et al	give an overview of the most mentioned smartphones’ pregnancy-related applications (Apps)	“BabyCenter my Pregnancy Today” was the most quoted app (39 times). The other two most mentioned applications were “Baby Bump” (30 times), designed to supply information about normal and abnormal symptoms during pregnancy, and “Pregnancy / Sprout”, mentioned 21 times

## Conclusion

Choosing a valid application to maintain pregnant women health and reduce traffic in the current situation will be very crucial. The aim of this article was choosing the best app, so it can be a suggestion for pregnant women and even gynecologists to introduce such apps despite the heavy traffic and technology era. Finally, the Amila app was selected as one of those apps that not only provides the required information to mothers, but also the ability to interact with doctors and specialists, due to its higher accuracy (%98), precision (%98) and usability than other apps(based on Table 4) clinical implications of this study and recommendations are as using more technologies with physicians and patients can help them monitor patients and themselves respectively, and can attached to wearable devices to aware them from disease or problems.

### Supplementary Information

**Additional file 1: Supplementary table 1.** Search strategy used for each online database.

## Data Availability

All data generated or analysed during this study are included in this published article and its supplementary information files.

## Abbreviations

App: Application
mHealth: Mobile health
MPH: Perinatal methylphenidate
