# Supplementary material for: Mobile health apps for pregnant women usability and quality rating scales: a systematic review
Source: BMC Pregnancy Childbirth. 2024 Jan 5;24:34. doi: 10.1186/s12884-023-06206-z (PMC10768378; doi:10.1186/s12884-023-06206-z)
Supplement: Supplementary file 1 — Additional file 1: Supplementary table 1. Search strategy used for each online database. [file 12884_2023_6206_MOESM1_ESM.docx]

| **Supplementary table 1. Search strategy used for each online database** |
| --- |
| ***PubMed*** |
| ((MOBILE APP [tiab] OR APP [tiab] OR mobile health[tiab] OR mHealth [tiab] OR Eggs[tiab]) AND (pregnancy [Mesh] OR pregnant women [tiab] OR pregnant [tiab]) |
| ***sciencedirect*** |
| ("MOBILE APP" OR "APP" OR "mHealth" OR "mobile health") AND ("pregnancy" OR "pregnant women" OR "pregnant") |
| ***Google Scholar*** |
| ("MOBILE APP" OR "APP" OR "Mhealth” OR "mobile health”) AND (“pregnancy " OR "pregnant women" OR "pregnant") |
